# Gene Delivery of Manf to Beta-Cells of the Pancreatic Islets Protects NOD Mice from Type 1 Diabetes Development

**DOI:** 10.3390/biom12101493

**Published:** 2022-10-16

**Authors:** Kailash Singh, Orian Bricard, Jeason Haughton, Mikaela Björkqvist, Moa Thorstensson, Zhengkang Luo, Loriana Mascali, Emanuela Pasciuto, Chantal Mathieu, James Dooley, Adrian Liston

**Affiliations:** 1 Immunology Programme, The Babraham Institute, Cambridge CB22 3AT, UK; 2 Department of Medical Cell Biology, Uppsala University, 751 05 Uppsala, Sweden; 3 VIB Center for Brain and Disease Research, KU Leuven—University of Leuven, 3000 Leuven, Belgium; 4 Department of Microbiology, Immunology and Transplantation, KU Leuven—University of Leuven, 3000 Leuven, Belgium; 5 Department of Neurosciences, KU Leuven—University of Leuven, 3000 Leuven, Belgium; 6 Department of Chronic Diseases and Metabolism, KU Leuven—University of Leuven, 3000 Leuven, Belgium

**Keywords:** type 1 diabetes, AAV, Manf, gene delivery, NOD mice, beta-cells

## Abstract

In type 1 diabetes, dysfunctional glucose regulation occurs due to the death of insulin-producing beta-cells in the pancreatic islets. Initiation of this process is caused by the inheritance of an adaptive immune system that is predisposed to responding to beta-cell antigens, most notably to insulin itself, coupled with unknown environmental insults priming the autoimmune reaction. While autoimmunity is a primary driver in beta-cell death, there is growing evidence that cellular stress participates in the loss of beta-cells. In the beta-cell fragility model, partial loss of islet mass requires compensatory upregulation of insulin production in the remaining islets, driving a cellular stress capable of triggering apoptosis in the remaining cells. The Glis3-Manf axis has been identified as being pivotal to the relative fragility or robustness of stressed islets, potentially operating in both type 1 and type 2 diabetes. Here, we have used an AAV-based gene delivery system to enhance the expression of the anti-apoptotic protein Manf in the beta-cells of NOD mice. Gene delivery substantially lowered the rate of diabetes development in treated mice. Manf-treated mice demonstrated minimal insulitis and superior preservation of insulin production. Our results demonstrating the therapeutic potential of Manf delivery to enhance beta-cell robustness and avert clinical diabetes.

## 1. Introduction

Diabetes is a disease of dysfunctional glucose regulation. The predominant forms of diabetes are type 1 diabetes (T1D) and type 2 diabetes (T2D), although there are a diverse set of additional forms, which include aspects of one or both diseases. In T1D, disease is initiated and caused by inheritance of an adaptive immune system that is predisposed to responding beta-cell antigens, most notably to insulin itself [1]. However, despite autoimmunity being effectively established by three years of age in over 80% of cases, most patients are diagnosed many years, or even decades, after establishment of persistent anti-islet autoimmunity [2]. There have been over 35 years of failed immunotherapeutic trials aimed at stopping the autoimmune response in T1D, suggesting that these final stages, after the initial autoimmunity, may not solely be autoimmune in nature but may also be due to an intrinsic beta-cell vulnerability or fragility to cell death.

In contrast to T1D, T2D is not an autoimmune disease. In the earliest pre-diabetic stages of the disease, normally insulin-responsive cells (such as hepatocytes) become resistant to insulin, with reduced signalling through the insulin receptor. The transition between pre-diabetes and early-stage T2D can be difficult to catch in a standard clinical setting and is, at this early stage, reversible [3], with pancreatic beta-cells initially able to compensate for insulin resistance by increasing the amount and duration of insulin secretion. Many patients remain in a grey area of diagnosis at this stage, where diet modification and anti-diabetogenic drugs are sufficient to avoid the prolonged hyper-glycemia that is the pathological outcome of untreated T2D. However, a sizable subset of early-stage T2D patients go on to develop a loss of beta-cell mass. A critical inflection point in the disease process is the point at which insulin levels (having escalated with increasing insulin resistance) start declining, with the loss in beta-cell numbers greater than the compensatory capacity of the remaining cells [4,5]. In this late-stage of T2D, insulin administration is required, but, due to the pre-existing insulin resistance, outcomes are poor [6]. The mechanistic basis of beta-cell decline during the early-to-late stage transition is highly controversial, with convincing arguments being put forward as to the role of glucose toxicity [6], endogenous beta-cell stress due to excessive insulin production [7], or inflammation-mediated destruction [8], among other plausible hypotheses.

The concept that primary beta-cell defects may lie at the heart of susceptibility to both T1D and T2D has been proposed by us [9,10] and others [11,12,13], with various iterations and degrees of emphasis on the differences or similarities between T1D and T2D. The beta-cell fragility model can be defined as one where variation in the intrinsic fragility or robustness of beta-cells contributes to the development of diabetes. Just as susceptibility to autoimmunity and insulin resistance varies across individuals, due to genetic and environmental influences, beta-cells vary across individuals in their ability to survive autoimmune or metabolic insults [10]. The presence of “fragile” beta-cells in an individual would sensitize for diabetes—either T1D, for individuals with autoimmune susceptibility, or T2D, for individuals with metabolic stress. By contrast, individuals with “robust” beta-cells would be more likely to remain at a pre-clinical stage with delayed diabetes development. Under this model, increasing the robustness of beta-cells would be an effective therapeutic strategy for individuals at high risk for either T1D or T2D, or to prevent further progression of T2D.

Support for the beta-cell fragility hypothesis comes from disease models that allow dissection of beta-cell-intrinsic function. The primary animal model of T1D is the non-obese diabetic (NOD) mouse, which hosts a large set of genetic polymorphisms increasing susceptibility to anti-islet autoimmunity [14]. Intriguingly, the NOD diabetes-associated loci overlap with diabetes-associated loci from the related T2D mouse strain, Nagoya-Shibata-Yasuda (NSY) mice [11], raising the possibility of a shared genetic predisposition. Our laboratory performed a molecular dissection of NOD genetic control over beta-cell viability and found strong evidence that the shared component of diabetes susceptibility may be dependent on beta-cell fragility. NOD mice possess variants in *Glis3* that have beta-cell-intrinsic functions, rendering the beta-cells highly susceptible to apoptosis [9]. When NOD mice are exposed to beta-cell stressors that mimic the compensatory insulin over-production observed in T2D, this beta-cell fragility is sufficient to tip the mice into overt diabetes, while the same stressors remain sub-clinical in mice with robust beta-cells [9]. The molecular mechanism by which the NOD *Glis3* variant enhances beta-cell susceptibility to apoptosis appears to be via reduced upregulation of mesencephalic astrocyte-derived neurotrophic factor (Manf), an obligate pro-survival factor for beta-cells [15]. Here we sought to increase the robustness of the beta-cells of the pancreas by correcting the downregulation of Manf present in NOD mice. Using an AAV-based gene delivery system, we drove expression of Manf in the beta-cells of NOD mice using the insulin promoter. Gene delivery of Manf substantially lowered the rate of diabetes development and insulitis in treated mice, proof-of-principle that correcting beta-cell fragility can avert clinical diabetes progression.

## 2. Materials and Methods

### 2.1. Mice

NOD mice were inbred and housed under semibarrier conditions in our animal facility, and fed a standard chow diet. Ten-week-old female NOD mice were used. Allocation to treatment group was made randomly at weaning, at the cage level. All experiments were performed in accordance with the University of Leuven Animal Ethics Committee guidelines. Sample sizes for mouse experiments were chosen in conjunction with the Animal Ethics Committee to allow for robust sensitivity without excessive use.

### 2.2. Diabetes Incidence Study

Mice were kept until 30 weeks of age and tested twice per week for glucose dysregulation by blood glucose and urine assessment with Diastix Reagent Strips (Bayer, Basel, Switzerland). Mice were diagnosed as diabetic when having glucosuria and a blood glucose (FreeStyle Freedom Lite, Abbott, Chicago, IL, USA) level over 250 mg/dL (13.9 mmol/L) for two consecutive readings. Glucose testing was performed on a blinded basis, with mice being coded by number until experimental end.

### 2.3. AAV Vector Production and Purification

AAV production was performed by VectorBuilder (Neu-Isenburg, Germany), using the classical tri-transfection method, with subsequent vector titration performed using a qPCR-based methodology [16,17]. For AAV8.*ins*-GFP and AAV8.*ins*-Manf, the mouse Manf coding sequence (accession number NM_029103.4) was cloned into a single stranded AAV8-derived expression cassette containing the 705 bp rat Insulin 2 promoter, woodchuck hepatitis post-transcriptional regulatory element (WPRE), and bovine growth hormone polyadenylation (bGH polyA) sequence. Control vectors were prepared by swapping the Manf coding sequence for that encoding-enhanced green fluorescent protein (EGFP, Vector Biolabs). Vector (100 µL total volume) was administered to mice via the interperitoneal route at 1 × 10^10^ vector genomes/dose. For AAV8.*glu*-GFP and AAV8.*glu*-Manf, mouse glucagon promotor was used.

### 2.4. Pancreatic Tissue Preparation for Morphological Analysis

After the pancreatic tissues were harvested, they were preserved and fixed in formaldehyde until the next step of tissue processing, which was washing. The washing was performed by rinsing the tissues in cold, running tap water for five–six hours. After washing, the paraffin embedding was performed overnight for 9 h and 30 min in the embedding machine (Thermo Scientific Microm STP 120 Spin Tissue Processor, Walldorf, Germany). During the first part of the embedding process, the pancreatic tissues were dehydrated by the addition of an increasing concentration of ethanol (Solveco, Rosersberg, Sweden) from 70% up to 99.6%. Following this, the pancreatic tissues were cleared through xylene (VWR Chemicals, Fontenay-sous-Bois, France) and at last immersed in paraffin (Merck KGaA Paraffin, Darmstadt, Germany). The following day, when the paraffin embedding was completed, the pancreatic tissues were assembled into paraffin wax blocks. The next step of the tissue processing was sectioning the pancreata into 5–6 μm thick sections. Sectioning was accomplished using a microtome (Thermo Scientific Microm HM355S Rotary Microtome, Walldorf, Germany) with a water bath containing distilled water with a temperature of 39–41 °C. Ten microscope slides (Epredia, SuperfrostTM Plus Adhesion Slides) were made from each of the paraffin-embedded tissue blocks, holding three sections per slide. The sections were made with little to no discard in between slides. After the sections were mounted onto the slides, they were placed to dry on a heating block set at 40 °C. Continuously throughout the sectioning process, the sections were fixed onto the slides at 70 °C for 1 h.

### 2.5. Immunohistochemical Staining for Insulin

Ten microscope slides were made from each mouse pancreas. Then, three of these slides (slide numbers 1, 6, and 10) were prepared for staining. Two different stainings were chosen: specific staining for insulin and a counterstaining with Haematoxylin. To prepare the samples for the insulin staining, deparaffinization was performed by placing the slides in xylene. Rehydration was then performed by first dipping the slides in the first container with ethanol and then incubating the slides for 5 min in the following one. The dipping and incubation were performed in multiple sets of containers, first in an ethanol series, with a decreasing concentration of ethanol (99.6%, 95%, 80% to 70%), and at last in distilled water. After the deparaffinization and rehydration, the slides were incubated for 10 min at room temperature in a hydrogen peroxide block solution (PBS-Tween and Hydrogen peroxide 30%, EMSURE^®^, Merck KGaA, Darmstadt, Germany) and then washed in PBS-Tween. Next, the sections were blocked in donkey serum (3% Normal Donkey Serum, Jackson ImmunoResearch Laboratories, Inc. Ely, England) for 30 min at room temperature. When the incubation was completed, the donkey serum was removed and then sections were stained with anti-insulin antibody dilution (1:1000 dilution in PBS containing 3% donkey serum) overnight at 4 °C. The following day, the slides were first washed in PBS-T for 3 × 5 min. Next, the sections were stained with donkey anti-guinea pig antibody (1:500, Peroxidase-conjugated AffiniPure Donkey Anti-Guinea pig IgG, Jackson ImmunoResearch Laboratories, Inc. Ely, England) for 60 min in the humidity chamber at room temperature. The tissues were then washed in PBS-T and then incubated in DAB solution (PBS-Tween and DAB + Hydrogen peroxide 30%, Merch KGaA, Darmstadt, Germany) for 4 min. After that, slides were dipped in distilled water a few times and then in Haematoxylin (Histolab products AB, Askim, Sweden) for 25 s. After being stained for Haematoxylin, the slides were washed under cold, running tap water for 20 min. Finally, for dehydration, the samples were dipped in an increasing concentration of ethanol. First, one time each in 70% and 80% ethanol, then two times each in 95% and 99.6% ethanol, and at last, the slides were incubated in xylene. Slides were then mounted using a mounter medium (Pertex^®^ Mounting Medium, Histolab Products AB, Askim, Sweden).

### 2.6. Histological Analysis

The slides were analysed in a blinded manner under a light microscope (Olympus BX53, Olympus Corporation, Tokyo, Japan). The degree of insulitis in the islets was graded as: (0) no immune cell infiltration, (1) peri-infiltration, (2) <⅓ infiltration, (3) >⅓ infiltration, or (4) no islet structure left. In addition, the insulin content in the islets was determined by the presence of brown colour from the DAB staining. The Langerhans islets were characterized as either insulin-positive (brown colour) or insulin-negative (no brown colour).

### 2.7. Immunohistochemical Staining for Glucagon, GFP, Insulin, Manf and Nuclei

To prepare the samples for the staining, deparaffinisation was performed as described in Section 2.6. After deparaffinisation, sections were blocked in donkey serum for 30 min at room temperature. Sections were stained with anti-glucagon (1:250 dilution; eBioscience, San Diego, CA, USA), anti-GFP (1:200; Life Technologies, Carlsbad, CA, USA) or anti-insulin (1:1000 dilution) and anti Manf (1:200 dilution) (Sigma-Aldrich) in 3% donkey serum overnight at 4 °C. This was followed by two washes and staining with Alexa-594 conjugated donkey anti-guinea pig antibody or Cy3-conjugated Streptavidin and Alexa 488 anti-rabbit antibodies for 60 min in the humidity chamber at room temperature. All the secondary antibodies were from Jackson ImmunoResearch Laboratories, Inc. (Ely, England) and diluted in 1:300 PBS containing 3% donkey serum. The nuclei were stained with DAPI (Life Technologies) for five minutes in the humidity chamber at room temperature. After two washes, slides were dried and mounted with Fluoromount-G (Southern Biotech Brimingham, AL, USA).

### 2.8. Confocal Imaging and Mean Fluorescence Intensity Analysis

Confocal imaging was performed using a laser caning confocal microscope Zeiss LSM 780 (Carl Zeiss, Jena, Germany). All images were analysed using Zeiss Zen Blue software. Mean fluorescence intensity was determined using ImajeJ software (NIH, Bethesda, MD, USA).

### 2.9. Enzyme-Linked Immunesorbent Assay

Serum samples were analysed to determine the insulin levels using Ultra-Sensitive Insulin ELISA kit (Mercodia, Uppsala, Sweden).

### 2.10. Statistical Analysis

The GraphPad Prism 9.4.0 Software (San Diego, CA, USA) was used for the statistical analysis of the data. First, the percentage of non-diabetic mice was analysed using a Kaplan–Meier graph. Kruskal–Wallis test followed by the Dunn’s test was performed for multiple comparisons. The data of the insulitis grading and insulin content in NOD mice are shown as mean ± SEM, and a *p*-value < 0.05 was considered to be a statistically significant difference between the observations.

## 3. Results

### 3.1. Beta-Cell Specific Gene Delivery of Manf Prevents Diabetes Development In Vivo

**Figure 1:**
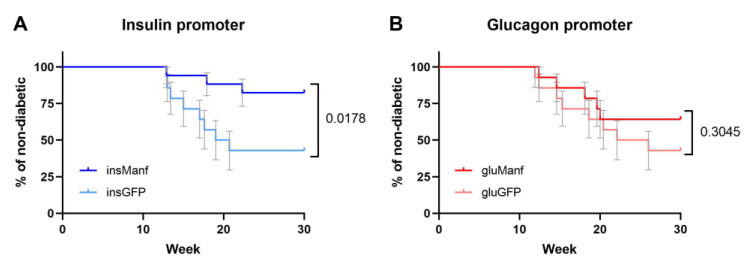
AAV8.*ins*-Manf prevented diabetes development in NOD mice. The percentage of non-diabetic NOD mice was controlled after the end of treatment at 30 weeks of age. (**A**) The AAV8.*ins*-Manf group (*n* = 17) and the AAV8.*ins*-GFP group (*n* = 14) had 82.4% and 42.8% non-diabetic mice, respectively (*p*-value of 0.0178). (**B**) The comparison between two groups with glucagon promoters, AAV8.*glu*-Manf (*n* = 14) and AAV8.*glu*-GFP (*n* = 14), had a *p*-value of 0.3045, which show no statistically significant difference. For comparison a Log-rank test was used.

The identified association between defective Manf production and beta-cell fragility suggested that excess Manf production could be protective in the context of diabetes [9]. We therefore developed a gene delivery-based therapeutic system to deliver Manf to the islets in vivo. An AAV gene delivery system was used to drive the endogenous production of Manf in the islets. AAV8 was used, as this serotype has tropism covering both pancreatic alpha- and beta-cells [18]. The AAV8 capsid was coupled with the rat insulin promoter (AAV8.*ins*-Manf) or the mouse glucagon promoter (AAV8.*glu*-Manf), to drive production in the beta- and alpha-cells, respectively. Pre-diabetic NOD mice, at 10 weeks of age, were treated with either AAV8.*ins*-Manf, AAV8.*glu*-Manf, AAV8.*ins*-GFP, or AAV8.*glu*-GFP vector, and monitored for diabetes development (Figure 1A). Only AAV8.*ins*-Manf treatment in NOD mice reduced the diabetes rate from 58% to 18% (*p* = 0.0178). AAV8.*glu*-Manf treatment was not as effective (Figure 1B), with no significant protective effect compared to the control vector. This partial effect of AAV8.*glu*-Manf, with a diabetes rate intermediate between the control vectors and the AAV8.*ins*-Manf vector, was, however, non-significant in both directions (Figure 1B, Supplementary Figure S1A). The two control vectors gave highly similar diabetes progression curves (Supplementary Figure S1B). Together, these data demonstrate an effective capacity for treatment with AAV8.*ins*-Manf to prevent diabetes in NOD mice.

**Figure 2:**
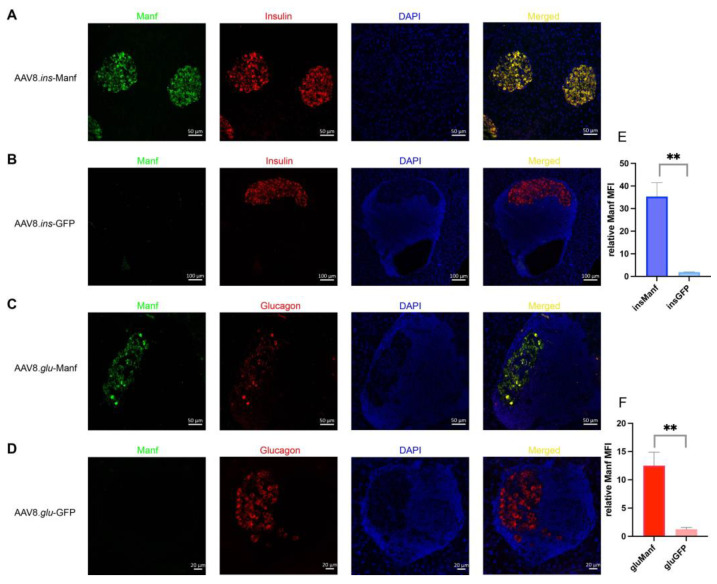
Islets of AAV8.*ins*-Manf- or AAV8.*glu*-Manf-treated mice express Manf. (**A**–**D**) AAV8.ins-Manf-, AAV8.ins-GFP-, AAV8.glu-Manf-, and AAV8.glu-GFP-treated mice were stained with antibodies: Manf (green), insulin (red), or glucagon (red) and DAPI (blue). (**E**,**F**) mean fluorescence intensity (MFI) of Manf in islets of AAV8.ins-Manf-, AAV8.ins-GFP-, AAV8.glu-Manf-, and AAV8.glu-GFP-treated mice. Three to four slides from each mouse (in total 4 mice per group) were stained with antibodies: Manf, insulin, or glucagon and DAPI. Confocal images were captured and analysed using ImageJ software for determining MFI. Results are presented in means ± SEM (*n* = 4 per group). Unpaired t-tests were performed for comparison, ** denote *p* < 0.05.

To confirm that the AAV8 vectors were transducing beta-cells and alpha-cells and driving cargo production, we assessed GFP production in the two control vector groups. Anti-GFP staining demonstrated that most beta-cells and alpha-cells were positive for GFP staining when using the AAV8.*ins* and AAV8.*glu* constructs, respectively (Supplementary Figure S2). Next, we investigated the expression of Manf in islets of mice treated with AAV8.*ins*-Manf, AAV8.*glu*-Manf, AAV8.*ins*-GFP, or AAV8.*glu*-GFP. Treatment with AAV8.*ins*-Manf drove a substantial upregulation of Manf, specifically within the beta-cell compartment (Figure 2A,E). A weaker effect was observed in the AAV8.*glu*-Manf vector (Figure 2C,F). These results indicate that the AAV8.*ins*-Manf vector is capable of efficient beta-cell specific gene delivery of Manf. In combination with the protective effect in diabetes, this suggests AAV8.*ins*-Manf may provide a blueprint for potential therapeutic use of gene delivery to increase islet robustness in diabetes patients.

### 3.2. Beta-Cell-Specific Gene Delivery of Manf Prevents Severe Insulitis in Islets In Vivo

**Figure 3:**
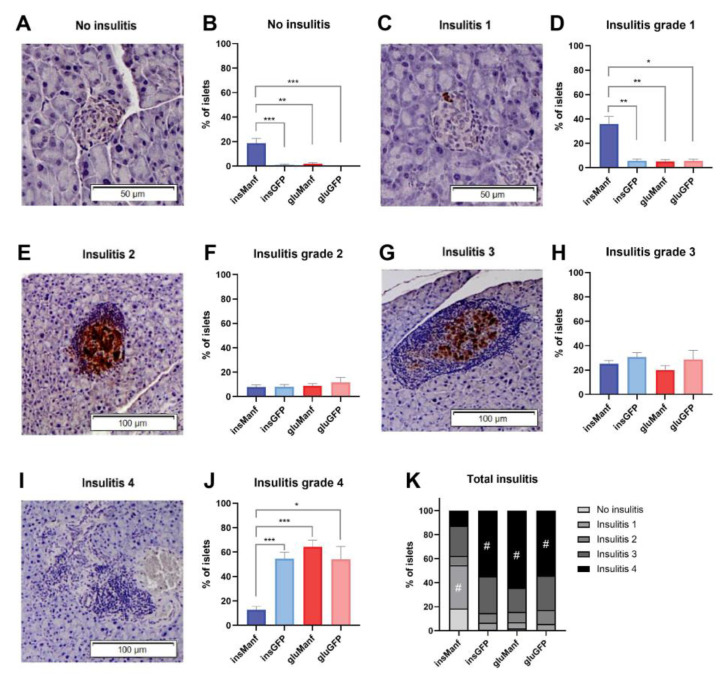
Beta-cell-specific gene delivery of Manf prevented islets from severe insulitis. Light microscope pictures of islets representing no insulitis (**A**), insulitis grade 1 (**C**), insulitis grade 2 (**E**), insulitis grade 3 (**G**), and insulitis grade 4 (**I**), are demonstrated as well as graphs of each insulitis grading, comparing the mean percentages of islets in each group (**B**,**D**,**F**,**H**,**J**). (**K**) Total insulitis. Results are presented in means ± SEM (*n* = 14–17/group). Kruskal-Wallis test, followed by the Dunn’s test was performed for multiple comparisons for finding the significant differences. *, ** and *** denote *p* < 0.05, *p* < 0.01 and *p* < 0.001, respectively.

Insulitis grading was performed to determine whether gene delivery of Manf could reduce insulitis in the pancreatic islets. The representative images of different insulitis grades are shown in Figure 3A,C,E,G,I. The mean percentage of islets was calculated for each insulitis grading. The data were analysed and compared between four treatment groups (AAV8.*ins*-Manf, AAV8.*glu*-Manf, AAV8.*ins*-GFP and AAV8.*glu*-GFP). The AAV8.*ins*-Manf group (total number of islets = 333) demonstrated the highest percentages of islets with no insulitis (15.3%) and with insulitis grade 1 (42.3%) (Figure 3B,D). For the insulitis grade 2, no differences were seen between the groups (Figure 3F). The AAV8.*ins*-GFP group (total number of islets = 461) displayed the highest mean percentage of islets of insulitis grade 3 (30.8%), although with no significance (Figure 3H). AAV8.*glu*-Manf (total number of islets = 248), AAV8.*glu*-GFP (total number of islets = 180), and AAV8.*ins*-GFP (total number of islets = 210) all had higher percentages of islets containing grade 4 insulitis compared with AAV8.*ins*-Manf (Figure 3J). The total insulitis in each group is shown in Figure 3K, and the insulitis grade with the highest mean percentage of islets for each group is marked (#).

### 3.3. Beta-Cell-Specific Gene Delivery of Manf Increases the Content of Insulin in Islets In Vivo

**Figure 4:**
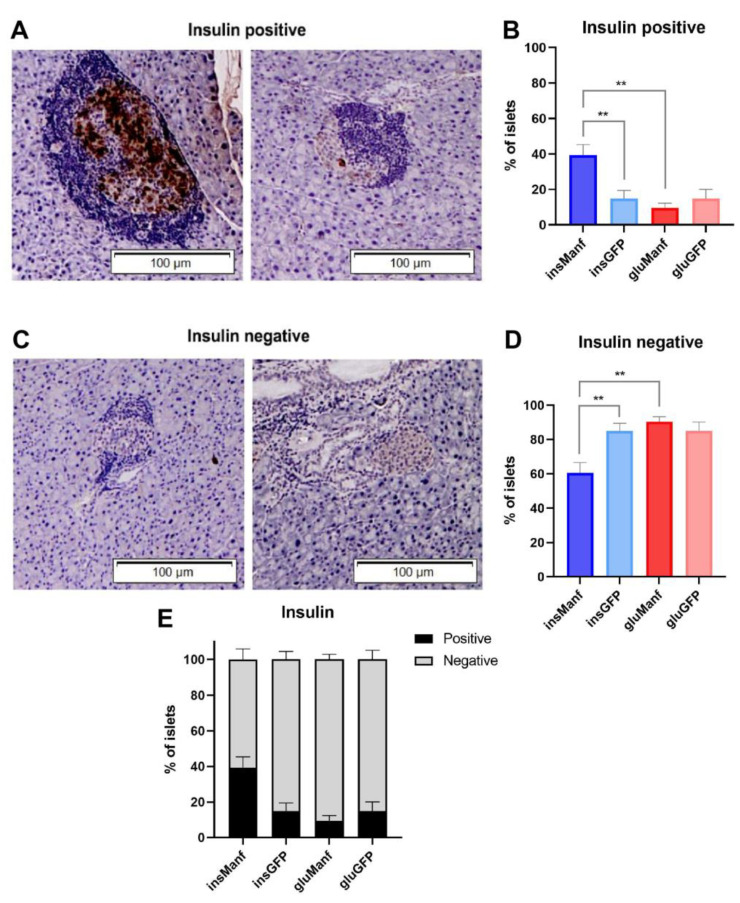
Beta-cell-specific gene delivery of Manf increased the content of insulin in islets of NOD mice. Light microscope pictures of NOD mice tissue sections immunohistochemically stained for insulin (**A**); a representative image of islets of AAV8.*ins*-Manf treated mice and (**C**); a representative image of islets of AAV8.*ins*-GFP treated mice) and graphs over the mean percentage of insulin-positive or insulin-negative islets in each group (**B**,**D**). (**A**) Two microscope pictures representing insulin-positive islets, where brown colour indicates insulin. (**B**) The percentages of insulin-positive islets in treated mice. (**C**) Two microscope pictures representing insulin-negative islets. (**D**) The percentages of insulin-negative islets in treated mice. (**E**) Percentages of insulin-positive and insulin-negative cells in total. Results are presented in means ± SEM (*n* = 14–17/group). Kruskal–Wallis test followed by Dunn’s test was performed for multiple comparisons. ** denote *p* < 0.01.

**Figure 5:**
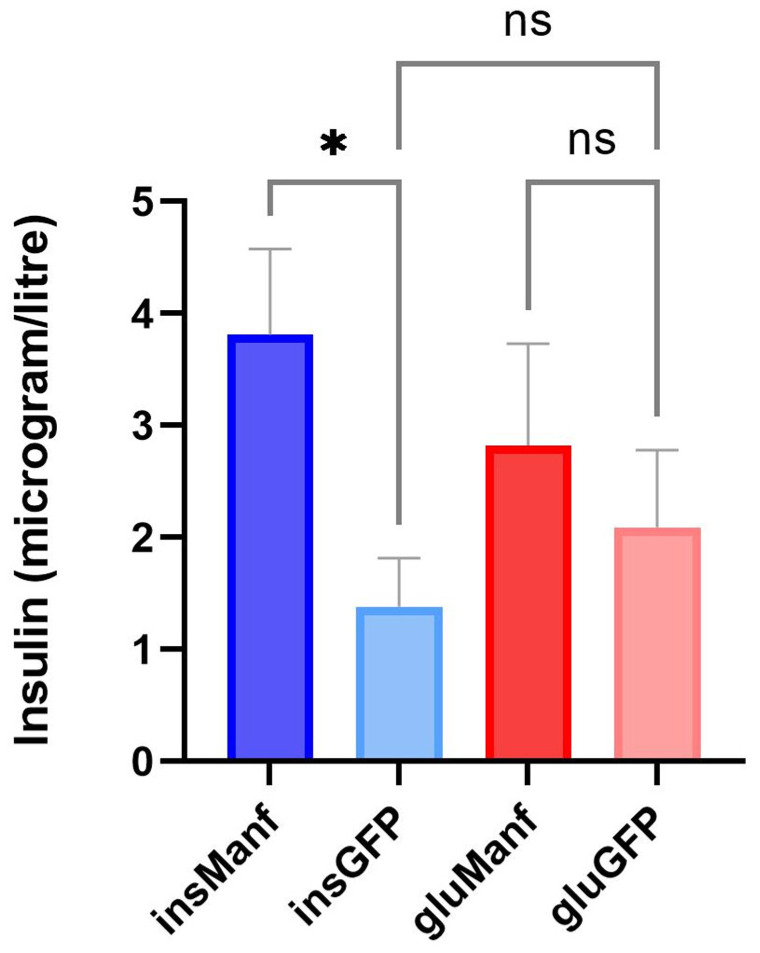
Beta-cell-specific gene delivery of Manf increased the serum levels of insulin in islets of NOD mice. Serum samples were analysed using Ultra-Sensitive Insulin ELISA kit. Results are presented in means ± SEM (*n* = 11–15/group). Kruskal–Wallis test followed by Dunn’s test was performed for multiple comparisons. * denotes *p* < 0.05.

Next, we determined the insulin content in pancreatic beta-cells of treated NOD mice using histological analysis. The insulin content in islets was determined to investigate if gene delivery of Manf could promote proliferation and viability of beta-cells in NOD mice. For analysing the insulin content in islets, no grading of the islets was done. Instead, the islets were classified as insulin-positive or insulin-negative, depending on whether bright brown colour cell/cells were visible under light microscope (Figure 4A,C and Supplementary Figure S3). A distinct bright brown colour could be seen in some islets, while only a bright brown dot could be spotted in others (Figure 4A). However, both of these islets were considered insulin-positive (Figure 4A). If no bright brown colour could be detected or if a light brown background was visible behind the islet, the islet was characterized as insulin-negative (Figure 4C). We found that 43.5% of islets in the AAV8.*ins*-Manf group (total number of islets = 333) were insulin-positive, compared to the AAV8.*ins*-GFP group (total number of islets = 461), where only 17.3% of islets were positive for insulin (Figure 4B). The AAV8.*glu*-Manf (total number of islets = 248) and the AAV8.*glu*-GFP (total number of islets = 180) groups had the lowest mean percentage of insulin-containing islets. In these groups, 12.5% and 15.0% of all islets were positive for insulin, respectively (Figure 4B). The AAV8.*glu*-Manf group had the highest mean percentage of insulin-negative islets (87.5%) (Figure 4D). However, both the AAV.*ins*-GFP and AAV8.*glu*-GFP groups also had higher mean percentage of insulin-negative islets as well, with 82.6% and 85% respectively (Figure 4D). The AAV8.*ins*-Manf group had the lowest mean percentage of insulin-negative islets (Figure 4D). The percentage of insulin-negative islets in the AAV8.*ins*-Manf group compared with the AAV8.*glu*-Manf and AAV8.*ins*-GFP groups displayed a significant difference (Figure 4D). To complement these data, we also determined the concentrations of insulin in serum samples of treated mice. Supporting a preservation of insulin production following beta-cell-specific Manf delivery, we found that the serum concentration of insulin was highest in mice treated with AAV8.*ins*-Manf (Figure 5).

## 4. Discussion

The Glis3-Manf pathway appears to be an important fulcrum for diabetes development. There are several lines of evidence indicating that GLIS3 is also a key anti-apoptotic mediator in humans. Using in vitro systems, exposure of human islets to certain dietary fats, such as palmitate and oleate, triggers apoptosis of beta-cells [19,20,21,22]. This effect is accompanied by a reduction in GLIS3 expression [23,24], and beta-cell apoptosis (in response to palmitate or inflammatory cytokines) is compounded by GLIS3 knockdown [13]. *GLIS3* polymorphism is linked to susceptibility to both T1D and T2D [25,26,27,28] as well as rare mutations also causing neonatal diabetes [29], demonstrating that expression variation can modify diabetes risk. In mouse models of beta-cell stress, decreased expression of Glis3 (from heterozygous status, or downstream of high fat diet exposure) sensitised to beta-cell death following islet stress [9].

Manf is a critical survival factor for pancreatic beta-cells, with Manf-deficient mice developing spontaneous diabetes due to beta-cell apoptosis [15]. Manf demonstrates one of the largest increases in expression following induction of the unfolded protein stress response [9], suggesting that it is a programmed stress-response pathway that enables continued survival. The same process is conserved in humans, with the addition of recombinant MANF protecting human pancreatic beta-cells from stress-induced apoptosis [30]. Evidence suggests that effective Manf upregulation during stress requires Glis3 expression. When Glis3 expression is impeded, either through genetic deficiency or diet-induced deficiency, Manf upregulation in response to stress is stunted [9]. Likewise, in human T2D islets, a positive relationship is observed between GLIS3 expression levels and MANF expression levels [9]. Together, this suggests that the anti-apoptotic effect of GLIS3 in human beta-cells may be mediated by MANF.

Here, we found that 82.4% NOD mice treated with AAV8.*ins*-Manf group did not develop diabetes, compared to the AAV8.*ins*-GFP group where 42.8% did not develop diabetes. This suggests that the ectopic expression of Manf in beta-cells is successful in preventing the development of diabetes in pre-diabetic female NOD mice. These results complement those reported by Lindahl et al., where an AAV-Manf delivery system was used to prevent streptozotocin-induced diabetes [15]. The Lindahl delivery system, however, used a strong ubiquitous promoter, which drives Manf in multiple cell types including in the brain [31], and required retrograde pancreatic duct injections to provide protection in a toxin-mediated model [15]. The results from our study demonstrate the ability of vector modification to impart beta-cell protection following intravenous delivery (at a 10-fold lower dose), and used spontaneous autoimmune diabetes in NOD mice, indicating the potential for MANF treatment to act as a therapeutic using more clinically viable approaches in the later stages of the physiological development of disease.

No significant difference could be seen in diabetic incidence rate between mice treated with AAV8.*glu*-Manf and the AAV8.*glu*-GFP group. This result could be explained, at a technical level, based on poor production of Manf by alpha-cells, although AAV8 is demonstrated to transduce alpha-cells [32] and AAV8-mediated delivery of a glucagon promoter-driven reporter results in efficient alpha-cell expression [33]. Assessment of Manf production indicated lower total cargo production within the islet using the *glu* promoter rather than the *ins* promoter, potentially supporting a technical explanation to this observation. A more intriguing possibility is that Manf must be expressed directly from beta-cells to prevent the development of diabetes. While Manf has been proposed to act as a soluble factor, the receptor for Manf is not identified, and an intracellular activity may be responsible for the protective effect. The histological analysis regarding the insulitis grading of the pancreatic islets illustrated that islets in the AAV8.*ins*-Manf groups had the lowest insulitis grading, which corresponds with previous studies that imply that Manf protects the beta-cells from destruction and prevents development of diabetes [15,34]. In line with this, we also found a higher content of insulin-positive cells in the islets of mice treated with AAV8.*ins*-Manf. This increased percentage of insulin-positive islets would enable insulin secretion to be maintained with a lower level of ER stress per cell, leading to healthier islets. In addition, due to the high content of insulin in this treatment group, it is possible that Manf not only protected against beta-cell loss, but also actively increased the number of beta-cells in the islets. This latter possibility is consistent with previous research demonstrating that Manf can increase the proliferation of beta-cells and the secretion of insulin [34], in addition to its more widely understood protective effect on apoptosis.

The use of an AAV-based gene delivery system raises the potential for translation to the human context. While viral vector-based therapeutics have had delayed uptake, improved safety profiles of modern vectors are driving a renaissance in gene delivery and gene therapy clinical trials, with nearly half of the currently open clinical trials based on AAVs. An improved robustness of beta-cells during cellular stress could be clinically beneficial in three different clinical contexts. First, and analogous to the NOD system used here, the system could be used to protect against T1D. Improved genetic and serology-based prediction may allow the identification of children pre-disposed to T1D for treatment prior to clinical onset. Alternatively, recent-onset T1D patients may be treated. The “honeymoon phase” that recent-onset T1D patients only enter following exogenous insulin treatment suggests that both remaining beta-cell mass is present, and also that the retained cells are operating in a sub-optimal manner due to excessive metabolic stress from insulin production. Increased robustness of these islets may prolong the honeymoon phase and reduce dependence on exogenous insulin. Second, the system could be used to improve islet transplantation. Transplanted islets have poor survival rates, and the transplantation process could be used as a window for exposure to AAV-based MANF buffering. Highly robust transplanted islets have the potential to increase long-term insulin production in patients. Third, the system could have utility in T2D patients. While initial stages of T2D are characterised by insulin resistance, ~30% of patients progress to insulin dependency, with beta-cell mass being reduced due to chronic metabolic stress. This insulin-dependent T2D stage is refractory to treatment, with a need for exogenous insulin coupled to insulin resistance, leading to high rates of secondary pathologies, such as diabetic foot or diabetic retinopathy. Treatment of early T2D patients at risk of progressing to insulin dependency, therefore, has the potential for high clinical impact in an at-risk population.

## 5. Conclusions

The results from this study indicate gene delivery of AAV8.*ins*-Manf prevents the development of diabetes in experimental T1D. The findings further indicate that expression of MANF in pancreatic beta-cells may have protective functions for survival and proliferation of beta-cells.

## Data Availability

Details of all experiments, including data and material used for performing this study, will be made accessible. Data are either included in the manuscript or available upon request.

## Supplementary Materials

The following supporting information can be downloaded at: https://www.mdpi.com/article/10.3390/biom12101493/s1, Figure S1: Neither the treatment groups, AAV8.*ins*-Manf and AAV8.*glu*-Manf, (P-value of 0.2533) nor AAV8.*ins*-GFP and AAV8.*glu*-GFP (P-value of 0.8862) showed a significant difference; Figure S2: Three to four slides of AAV8.ins-Manf (n = 3), AAV8.ins-GFP (n = 3), AAV8.glu-Manf (n = 3) and AAV8.glu-GFP (n = 3) treated mice were stained with antibodies; anti-GFP (green), insulin (red) or glucagon (red), and DAPI (blue); Figure S3: Representative images of pancreatic sections of AAV8.ins-Manf, AAV8.ins-GFP, AAV8.glu-Manf, and AAV8.glu-GFP treated mice. Brown color staining indicates insulin-positive cells.
