# Supplementary material for: Gene Delivery of Manf to Beta-Cells of the Pancreatic Islets Protects NOD Mice from Type 1 Diabetes Development
Source: Biomolecules. 2022 Oct 16;12(10):1493. doi: 10.3390/biom12101493 (PMC9599570; doi:10.3390/biom12101493)
Supplement: Supplementary file 1 [file biomolecules-12-01493-s001.zip › biomolecules-1917505-supplementary.pdf]

Supplementary Material

Gene Delivery of Manf to Beta-Cells of the Pancreatic Islets Protects NOD Mice from Type 1 Diabetes Development

Kailash Singh [1,2,*], Orian Bricard [1], Jeason Haughton [3,4], Mikaela Björkqvist [2], Moa Thorstensson [2], Zhengkang Luo [2], Loriana Mascali [3,4,5], Emanuela Pasciuto [3,4,5], Chantal Mathieu [6], James Dooley [1,†,*] and Adrian Liston [1,3,4,†,*]

[1] Immunology Programme, The Babraham Institute, Cambridge CB22 3AT, UK
[2] Medical Cell Biology, Uppsala University, Uppsala 751 05, Sweden
[3] VIB Center for Brain and Disease Research, KU Leuven-University of Leuven, Leuven 3000, Belgium
[4] Department of Microbiology, Immunology and Transplantation, KU Leuven—University of Leuven, Leuven 3000, Belgium
[5] Department of Neurosciences, KU Leuven—University of Leuven, Leuven 3000, Belgium
[6] Department of Chronic Diseases and Metabolism, KU Leuven—University of Leuven, Leuven 3000, Belgium
† These authors contributed equally to this work.
* Correspondence: kailash.singh@mcb.uu.se (K.S.); james.dooley@babraham.ac.uk (J.D.); al989@cam.ac.uk (A.L.)

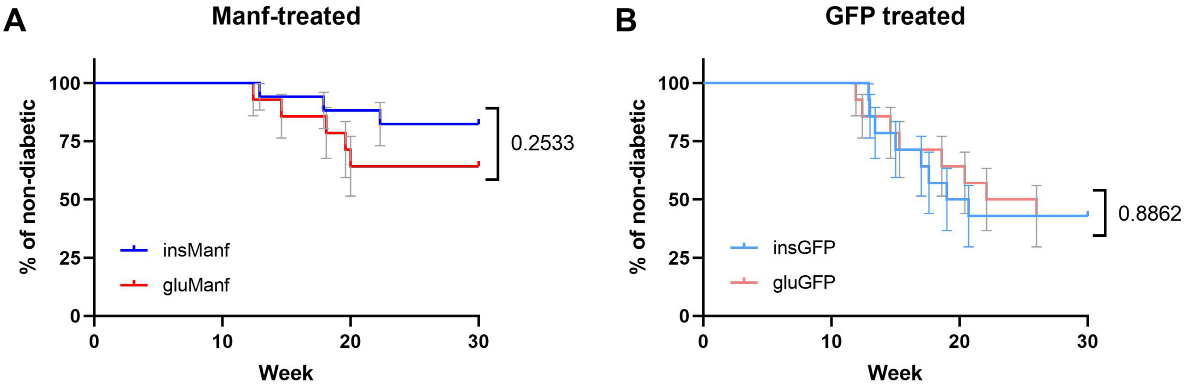

Supplementary Figure S1. Neither the treatment groups, AAV8.*ins*-Manf and AAV8.*glu*-Manf, (P-value of 0.2533) nor AAV8.*ins*-GFP and AAV8.*glu*-GFP (P-value of 0.8862) showed a significant difference.

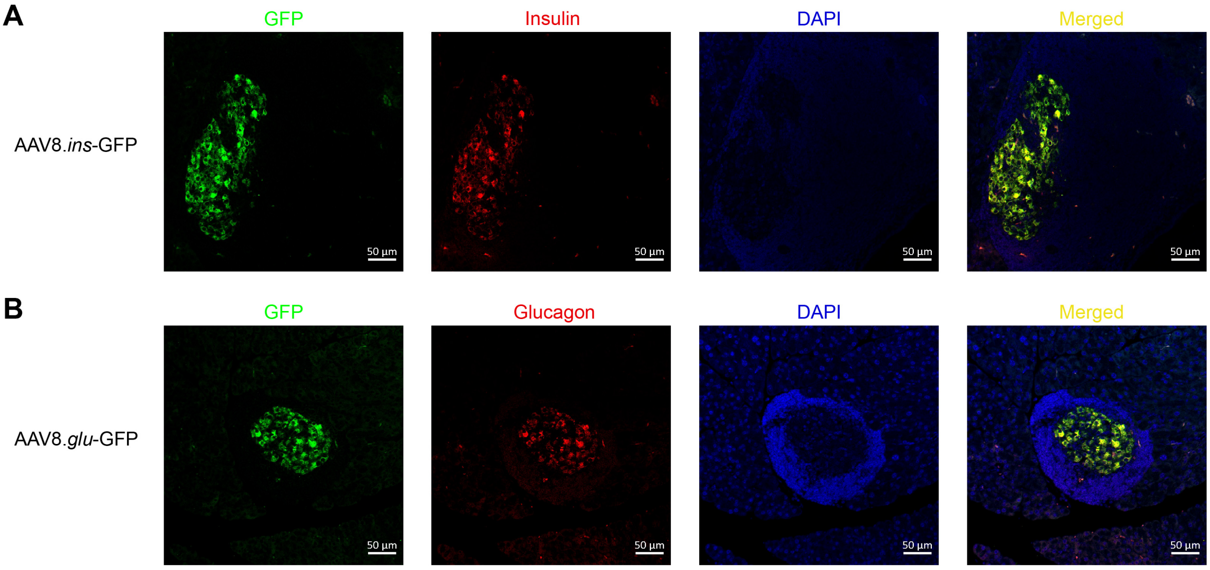

Supplementary Figure S2. Three to four slides of AAV8.ins-Manf (n=3), AAV8.ins-GFP (n=3), AAV8.glu-Manf (n=3) and AAV8.glu-GFP (n=3) treated mice were stained with antibodies; anti-GFP (green), insulin (red) or glucagon (red), and DAPI (blue).

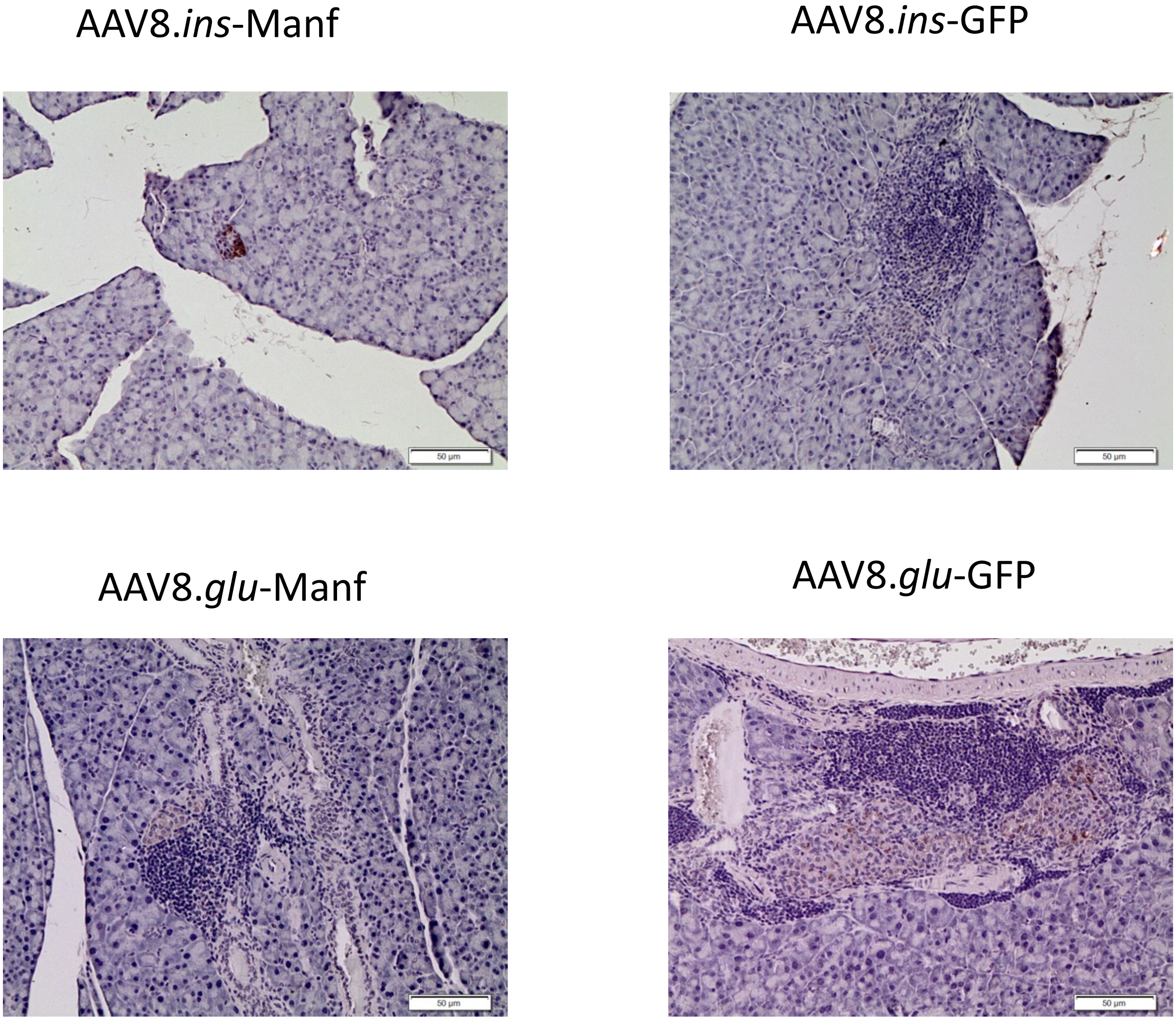

Supplementary figure S3. Representative images of pancreatic sections of AAV8.ins-Manf, AAV8.ins-GFP, AAV8.glu-Manf, and AAV8.glu-GFP treated mice. Brown color staining indicates insulin-positive cells.
